# Evolutionary transfer optimization-based approach for automated ictal pattern recognition using brain signals

**DOI:** 10.3389/fnhum.2024.1386168

**Published:** 2024-07-11

**Authors:** Piyush Swami, Jyoti Maheshwari, Mohit Kumar, Manvir Bhatia

**Affiliations:** ^1^Section for Visual Computing, Department of Applied Mathematics and Computer Science, Technical University of Denmark, Kongens Lyngby, Denmark; ^2^Danish Research Centre for Magnetic Resonance, Centre for Functional and Diagnostic Imaging and Research, Copenhagen University Hospital – Amager and Hvidovre, Copenhagen, Denmark; ^3^Biomedical Engineering Techies, Broendby, Denmark; ^4^School of Behavioural Forensics, National Forensic Sciences University, Gandhinagar, Gujarat, India; ^5^School of Electronics Engineering, VIT-AP University, Amaravati, India; ^6^Neurology and Sleep Centre, New Delhi, India

**Keywords:** electroencephalography, evolutionary transfer optimization, evolutionary multi-objective optimization, non-dominated sorting genetic algorithm, ictal pattern, epilepsy diagnosis

## Abstract

The visual scrutinization process for detecting epileptic seizures (ictal patterns) is time-consuming and prone to manual errors, which can have serious consequences, including drug abuse and life-threatening situations. To address these challenges, expert systems for automated detection of ictal patterns have been developed, yet feature engineering remains problematic due to variability within and between subjects. Single-objective optimization approaches yield less reliable results. This study proposes a novel expert system using the non-dominated sorting genetic algorithm (NSGA)-II to detect ictal patterns in brain signals. Employing an evolutionary multi-objective optimization (EMO) approach, the classifier minimizes both the number of features and the error rate simultaneously. Input features include statistical features derived from phase space transformations, singular values, and energy values of time–frequency domain wavelet packet transform coefficients. Through evolutionary transfer optimization (ETO), the optimal feature set is determined from training datasets and passed through a generalized regression neural network (GRNN) model for pattern detection of testing datasets. The results demonstrate high accuracy with minimal computation time (<0.5 s), and EMO reduces the feature set matrix by more than half, suggesting reliability for clinical applications. In conclusion, the proposed model offers promising advancements in automating ictal pattern recognition in EEG data, with potential implications for improving epilepsy diagnosis and treatment. Further research is warranted to validate its performance across diverse datasets and investigate potential limitations.

## 1 Introduction

### 1.1 Background

Epilepsy, a neurogenic disorder characterized by abrupt and transient disturbances in the body, manifests through sudden electrical bursts within the brain. These recurrent electrical discharges are commonly referred to as “epileptic seizures” or “ictal events” and more colloquially as “fits.” According to the survey, more than 50 million people worldwide are affected by epilepsy, representing approximately 2% of the global population (Moshé et al., 2015; World Health Organization, 2024). Consequently, the diagnosis of epilepsy is one of the utmost concerns. The most prevalent and reliable method for diagnosing epilepsy to date is recording the brain signals, primarily electroencephalography (EEG). Electrocorticography (ECoG) is also important and is particularly used for surgical intervention. Brain signals are primarily visually inspected by trained neuro-clinicians or neurophysiologists (Banerjee et al., 2009; Duque-muñoz et al., 2014). However, even standard EEG recordings for diagnosing epilepsy can last between 30 min to 6 h, rendering the visual scrutinization process very time-consuming. EEG data are often contaminated by motion artifacts, background noise, and interfering patterns from other neurological disorders. In developing countries, where the availability of neurophysiologists is low, diagnosing epilepsy using EEG becomes even more challenging and prone to manual errors (Banerjee et al., 2009). This situation makes the diagnosis of epilepsy using EEG very difficult and increases the likelihood of manual errors (Schuyler et al., 2007; Gandhi et al., 2010, 2012). Misdiagnosis of epilepsy often leads to the administration of improper drugs, which can prove to be life-threatening for patients. Hence, there is a dire need to develop an accurate, computationally fast, and robust tool for the diagnosis of epilepsy.

### 1.2 State-of-the-art

Significant research efforts have been dedicated to developing expert systems for the automated detection of ictal patterns or epileptic seizures in EEG. This section discusses some of the key findings reported in this area. Early breakthrough studies, such as those by Gotman (1999), relied on mimetic techniques that utilized descriptions provided by experienced neurophysiologists, including attributes such as crest, sharpness measures, time durations, inclinations, and more. However, this method proved to be inaccurate due to the heterogeneity among ictal patterns. Subsequent automated diagnosis methods for epilepsy involved the application of various frequency-domain techniques, such as the fast-Fourier transform (Polat and Güneş, 2007), and time-domain techniques, such as the empirical mode decomposition (Sharma and Pachori, 2015). Approaches based on FFT failed to capture the correct onset of ictal events due to their assumption of EEG as stationary despite its original non-stationary nature. The introduction of time–frequency domain techniques such as the short-time Fourier transform (Tzallas et al., 2009), especially wavelets (Gandhi et al., 2011; Swami et al., 2014; Faust et al., 2015; Edakawa et al., 2016; Swami et al., 2016a), aided in the development of many automated seizure detection models (Acharya et al., 2013).

Researchers have explored a wide variety of features for characterizing ictal patterns in EEG, including combinations of lower- and higher-order statistical parameters such as standard deviation (Gajic et al., 2015), kurtosis (Acharya et al., 2013), chaotic parameters such as correlation dimension and Lyapunov exponents (Tzallas et al., 2009; Acharya et al., 2013), Shannon entropy (Gandhi et al., 2011; Swami et al., 2017), energy (Swami et al., 2014, 2016a), and many more. Earlier, there was a general assumption that increasing the number of feature sets would improve the machine learning (ML) model’s accuracy. However, ample evidence suggests that increasing the dimensionality of the feature matrix could increase the computational cost of the expert system, while some features may even decrease the accuracy of the ML model. Hence, feature engineering for epilepsy diagnosis can present a paradox. Consequently, many researchers opt to exclusively utilize deep learning (DL)-based methods. While these methods perform optimally when trained with sufficiently large annotated/synthetic datasets (Pascual et al., 2020; Srinivasan et al., 2023; Dash et al., 2024), practical applications often encounter scarcity of such datasets and/or face challenges with the “black box” nature of the model. This opacity seldom instills confidence in clinicians to adopt new computer-aided diagnosis (CAD) systems. Therefore, identifying the underlying issues hindering the adoption of CAD in clinical settings is paramount.

### 1.3 Identification of problem statement and novelty

Some seminal research efforts on optimal feature selection have yielded noteworthy results (Gandhi et al., 2012). However, the majority of research endeavors focusing on feature optimization and selection have centered around a single objective function (Acharya et al., 2013; Dhiman and Saini, 2014; Dash et al., 2024), primarily aimed at enhancing the accuracy of expert systems. This often results in the development of models with either exceedingly slow computation times and high accuracy or rapid models with lower accuracy. Feature selection methods could provide a faster alternative (Nara et al., 2016; Krishnan et al., 2024); however, those methods usually do not solve multiple objectives. Additionally, the scalability issues of these models are frequently overlooked and often lead to the selection of a maximum number of features. This is an important issue for realizing practicability in clinical settings (Swami et al., 2018; Tirumani et al., 2018). A compromise between sensitivity and specificity rates has often been observed (Mormann et al., 2007; Swami et al., 2016a), rendering the replication and practical application of results in clinical settings challenging. The scarcity of extensive, annotated datasets further exacerbates these challenges. This research seeks to bridge the gaps between these extremes.

Moreover, much of the literature in epilepsy research lacks a clear delineation of the procedure for constructing optimization functions, hindering future replicable research. The present study aims to demonstrate the application of evolutionary transfer optimization (ETO) (Tan et al., 2021) through an evolutionary multi-objective optimization (EMO) approach. The transfer optimization methodology is employed to train specific datasets, with testing datasets consisting entirely of out-of-sample signals. In this context, the EMO method employed is the non-dominant sorting genetic algorithm (NSGA)-II, aimed at simultaneously minimizing the number of feature sets and error rates. The knowledge transfer is directed toward minimizing classification error rates while maximizing accuracy and minimizing features, aligning with the concept of Maximizing Accuracy while Minimizing Features (MAMF). This concept can also be applied to address a broad spectrum of not only neurological but also various real-life challenges.

### 1.4 Brief about the next sections

The following section of this article outlines the materials and methods employed. It comprehensively details the datasets utilized in this study and endeavors to present a procedural execution methodology for developing an expert system. Subsequently, the subsequent section of this article presents the results and discussion. Finally, the conclusions section summarizes the significant developments from this study and discusses its future scope.

## 2 Methods

### 2.1 Datasets

Datasets from three different repositories were used in this study. The first dataset is freely available in the epilepsy EEG repository of the University of Bonn (UoB) (Andrzejak et al., 2001). The datasets within this repository have become a common benchmark for validating expert systems for detecting epileptic seizures. The datasets considered from this database are named set C, set D, and set E. Each of these subsets consists of intracranial EEG, i.e., electrocorticography (ECoG) segments acquired with a sampling rate of 173.61 Hz from five epilepsy patients, with each segment comprising 4,097 samples lasting for a duration of 23.6 s. The signals in set C were acquired from the region around the hippocampus location opposite the hemisphere of the epileptogenic zone, while the signals in set D were acquired from the epileptogenic zone. Both sets C and D consisted of interictal (non-ictal) events, whereas only the signals in set E consisted of epileptic seizure (ictal) events.

The second dataset considered in this study is available from our repository (Swami et al., 2019). The signals in this repository were collected from 10 epilepsy patients using the Grass Telefactor Comet AS40 machine. The acquisition was conducted at the Neurology & Sleep Centre (NSC) by a trained clinician under the supervision of a neurophysiologist. During acquisition, gold-plated scalp EEG electrodes were positioned according to the international 10–20 electrode placement system. The data collected at 200 Hz from all channels were segmented into signals lasting for a duration of 5.12 s, comprising 1,024 samples. The subsets named interictal and ictal events were considered in this study.

The third dataset considered in this study was collected from the database of Sri Ganga Ram Hospital (SGRH). The signals downloaded from this repository were collected from 20 epilepsy patients (Gandhi et al., 2011, 2012; Swami et al., 2016a). The sampling rate during acquisition was fixed at 400 Hz, and the Grass Telefactor Twin3 EEG machine was used for acquisition. The data from all channels were segmented into signals lasting for a duration of 10 s, comprising 4,000 samples. The subsets with interictal and ictal stages were considered from this database.

**Figure 1:**
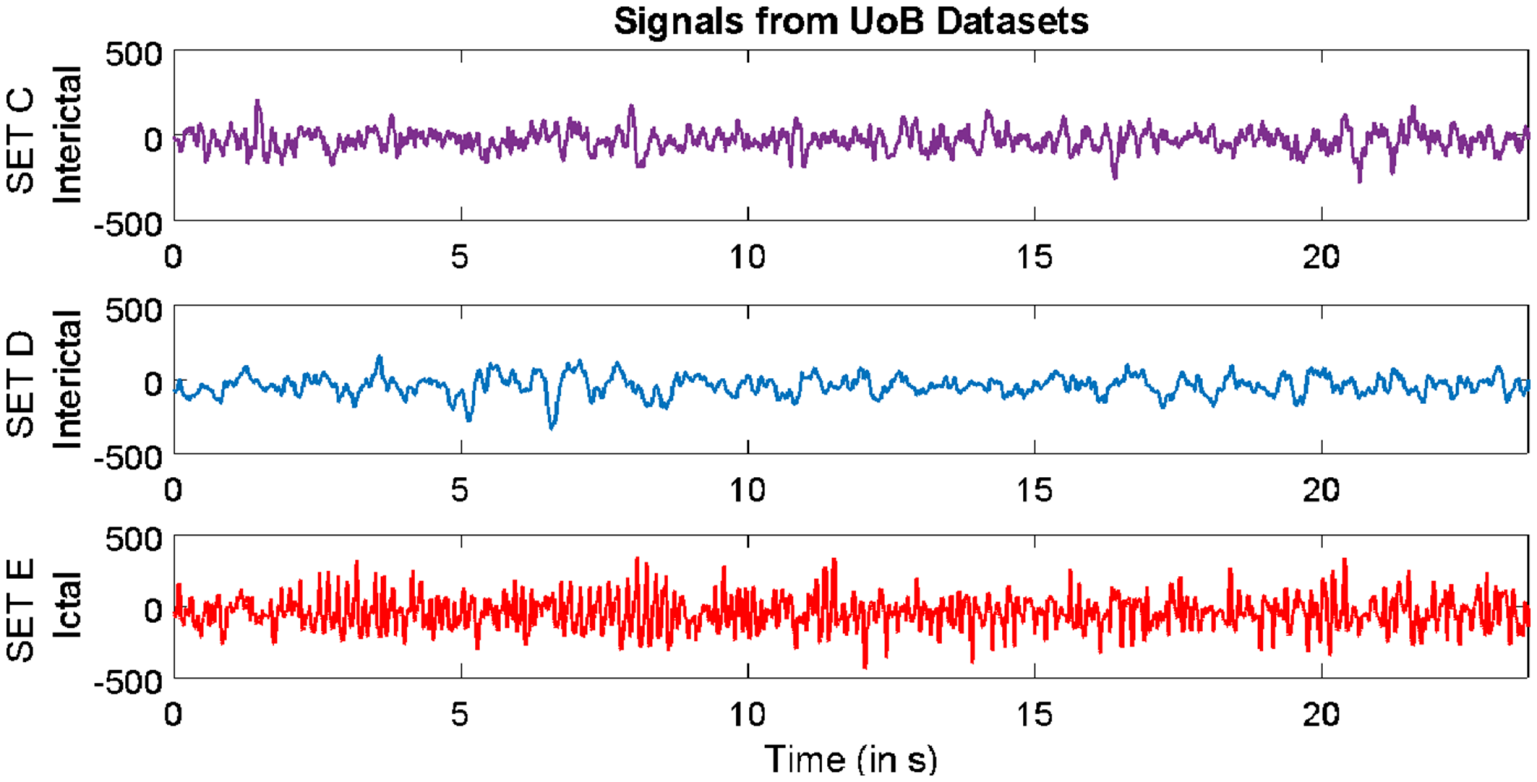
Example of signals from University of Bonn (UoB) datasets.

**Figure 2:**
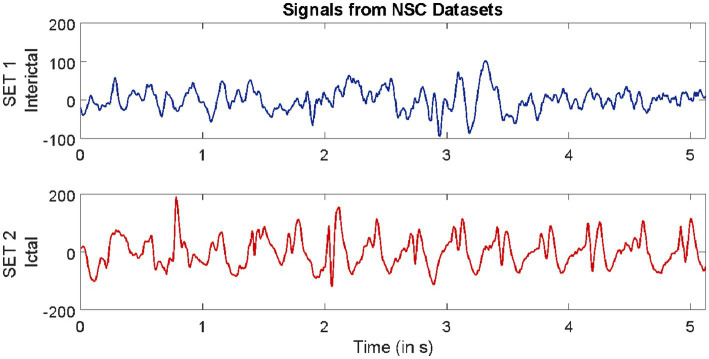
Example of signals from Neurology & Sleep Centre (NSC) datasets.

**Figure 3:**
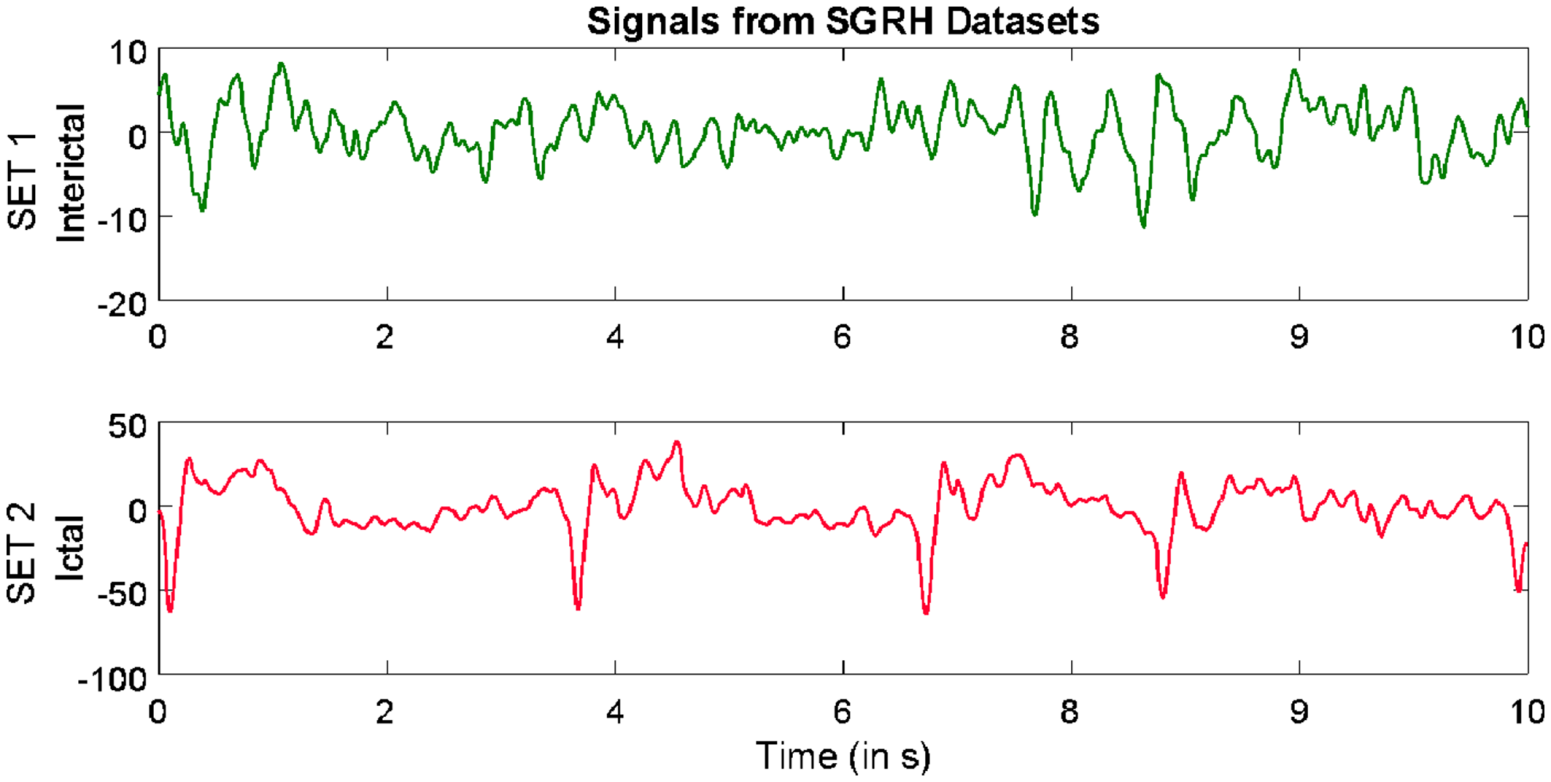
Example of signals from Sri Ganga Ram Hospital (SRGH) datasets.

Samples of EEG segments from each of the three repositories are shown in Figures 1–3.

### 2.2 Feature engineering

The process involves utilizing domain knowledge of the signals/datasets to extract a relevant set of attributes, referred to as features, which can then be fed into the ML classifier. The entire feature engineering procedure of this study is outlined as follows.

#### 2.2.1 Multi-resolution analysis (MRA) using wavelet packet transform (WPT)

**Figure 4:**
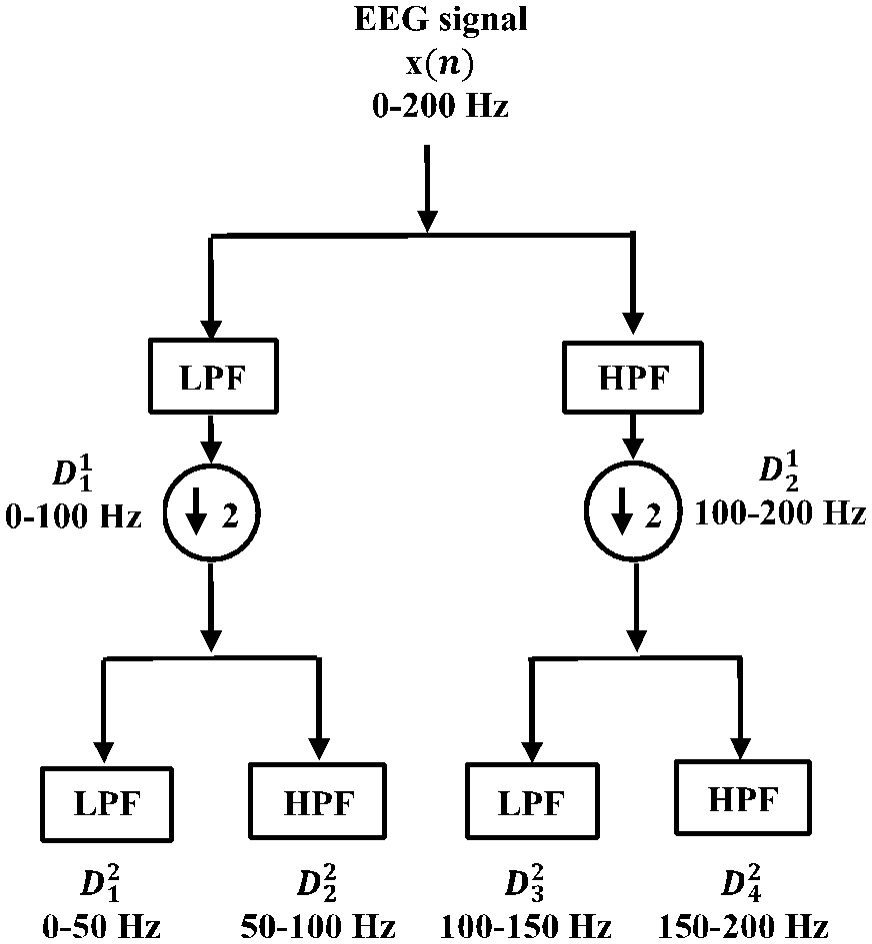
Double-branched architecture of wavelet packet transform (WPT).

This involves selecting the most relevant wavelet transforms that could completely characterize the signal. The wavelet coefficients $W_{i}$ with subspaces $S_{i}⊥W_{i}$ , i∈Z satisfying the multi-resolution analysis (MRA) conditions. Unlike discrete wavelet transform (DWT), the double-branched architecture of wavelet packet transform (WPT) provides a much smaller separation between the frequency bands and aids finer analysis. This technique has proven effective over DWT (Swami et al., 2017). The wavelet coefficients of the last decomposition level were stored for extracting features. For signal $x\left(n\right)$ , an orthonormal basis of $W_{i}$ can be given by $\Psi \left(n\right)$ , which is controlled by time shift and dilation parameters. Based on our previous findings (Gandhi et al., 2011), “Coiflets” mother wavelet with a single scaling function was selected in this study. The MRA using WPT was adopted in this study. In this method, a signal $x\left(n\right)$ is passed through a series of quadrature mirror filters (Gandhi et al., 2012; Swami et al., 2015a, 2017). During this recursive process, details and approximations are fed into the next filters. The double-branched architecture of WPT is shown in Figure 4. As an example, the input signal with a sampling rate $F_{s}=200$ Hz is fed into the WPT architecture in Figure 4. Resampling all input signals to a uniform frequency guarantees that the feature extraction is conducted consistently across signals with identical spectral characteristics (Frølich et al., 2015). Thereby also ensuring knowledge transfer. After the signal is fed into the WPT architecture, the frequency band for the input signal range between $\left[0-\left(F_{s}/2\right)\right]$ Hz. If we denote the wavelet coefficient by $W_{f}^{l}$ where, l is the decomposition level and f is the index of the frequency band, then the signals are decomposed/downsampled by 2 into the details $W_{1}^{1}$ (i.e., approximation with frequency band between $\left[0-\left(F_{s}/4\right)\right]$ Hz) and $W_{2}^{1}$ (i.e., detail with frequency band between $\left[\left(F_{s}/4\right)-\left(F_{s}/2\right)\right]$ Hz after the first decomposition level). Similarly, after l = 2, the preceding $W_{1}^{1}$ is decomposed into $W_{1}^{2}$ (i.e., approximation with frequency band between $\left[0-\left(F_{s}/8\right)\right]$ Hz) and $W_{2}^{2}$ (i.e., detail with frequency band between $\left[\left(F_{s}/8\right)-\left(F_{s}/4\right)\right]$ Hz). In addition, the $W_{2}^{1}$ is decomposed into $W_{3}^{2}$ (i.e., approximation with frequency band between $\left[\left(F_{s}/4\right)-\left(3F_{s}/8\right)\right]$ Hz) and $W_{4}^{2}$ (i.e., approximation with frequency band between $\left[\left(3F_{s}/8\right)-\left(F_{s}/2\right)\right]$ Hz). This process was recursively continued till seventh decomposition level, thus generating $2^{l}$ = 128 wavelet coefficients. It is very important that the length of the signal is sufficient to capture the ictal or non-ictal pattern (Behara et al., 2016).

#### 2.2.2 Features derived from phase space representations (PSRs)

The visualization of phase space representations (PSRs) is useful for studying the dynamics and state of biomedical signals such as EEG (Sharma and Pachori, 2015; Swami et al., 2015b,c; Anuragi et al., 2022). The 3D PSRs are calculated by using equation (1).


(1) $$\begin{array}{l} \mathrm{Phase}\;\mathrm{Space}\;\mathrm{Representations}\;PSR_{m}\\ \;=\left[V_{m},V_{\left(m+\tau \right)},\ldots ,V_{\left\{m+2\tau \right\}}\right] \end{array}$$

where, V represents EEG vectors of signal $x\left(n\right)$ , τ represents the time lag, m=1,2,…,M−2τ , with total number of data points M . EEG signals are from elliptical paths, which are more irregularly shaped for ictal patterns (Swami et al., 2016b). The irregularities in the elliptical paths were quantified by evaluating Euclidean distances between the delayed vectors using equation (2).


(2) $$\mathrm{Euclidean}\;\mathrm{distances}\;ED_{m}=\sqrt{\left({V_{m}}^{2}+{V_{m+1}}^{2}+{V_{m+2}}^{2}\right)}$$

To highlight the differences between the Euclidean distances of the non-ictal versus ictal patterns in EEG signals, statistical features such as standard deviation $SD_{pq}$ or $F_{1}$ (given by equation 3) and range (i.e., the difference between the maximum and minimum values) $RN_{pq}$ or $F_{2}$ were calculated. Here, p is the number of segmented EEG signals and q is the number of wavelet coefficients (fixed to 128).


(3) $$\begin{array}{l} \mathrm{Standard}\;\mathrm{deviation}\;\mathrm{values}\;\left(SD_{pq}\right)\\ \;=\sqrt{\left[\frac{1}{Q-1}\overset{Q}{\underset{q=1}{\sum }}{\left(\omega_{pq}-\mu_{pq}\right)}^{2}\right]} \end{array}$$

The $SD_{pq}$ and $RN_{pq}$ are computed from all the 128 coefficients and considered as features for classification tasks.

#### 2.2.3 Singular value decomposition (SVD) features or $F_{3}$

The singular value decomposition (SVD) is a tool for decomposing a matrix into its Eigenvalues, which is suitable for a non-square matrix. Hence, it is a useful measure for extracting the algebraic properties from large data such as EEG to study its dynamics. The SVD covariance matrix C is given by equation (4).


(4) $$SVD=\mathrm{US}V^{T}$$

Where singular values $S=\mathrm{diag}\left[\sigma_{1,}\sigma_{2},\sigma_{3},\ldots ,\sigma_{N},0,\ldots 0\right]$ consists of a diagonal matrix with singular values $\sigma_{1,}\sigma_{2},\sigma_{3,}\ldots ,\sigma_{N}$ , while, $U=\left[u_{1,}u_{2},u_{3},\ldots ,u_{i}\right]\in R^{i\times i}$ and $V=\left[v_{1,}v_{2},v_{3},\ldots ,v_{j}\right]\in R^{j\times j}$ represent unitary matrices. The singular values were calculated for each coefficient of the last decomposition level, and the final matrix is denoted by $SVD_{pq}$ . Hence, 128 singular values are considered for classification.

#### 2.2.4 Energy features or $F_{4}$

The abrupt neuronal discharges during the episodes of epileptic seizures consume high energy levels of the brain. This creates a misbalance between the energy levels within the brain. Hence, the evaluation of energy (EN) features directly from the wavelet coefficients allows us to quantify the difference between the energy levels during non-ictal and ictal events (Swami et al., 2016a). In this study, EN features are computed from all 128 coefficients and denoted as $EN_{pq}$ .

Finally, the complete feature matrix was formed by the horizontal concatenation of all the 512 (128 coefficients × 4) features given by equation (5).


(5) $$F_{pq}=\left[SD_{pq}RN_{pq}SVD_{pq}EN_{pq}\right]=\left[F_{1}F_{2}F_{3}F_{4}\right]$$

Where p is the index for EEG segments, q is the index for features, and it equals 1, 2, 3, …, 512. For the selection of the optimum number of features, the entire feature matrix $F_{pq}$ was provided as input to the evolutionary multi-objective optimization model.

### 2.3 Evolutionary multi-objective optimization (EMO) using non-dominated sorting genetic algorithm (NSGA)-II

The GA is an evolutionary computing algorithm that is based on biological evolution. In multi-objective GA, more than one objective is optimized simultaneously to achieve the best-compromised solution (Deb, 2001; Smith, 2002). In this study, we have used the NSGA-II method for minimization of the number of required features and the error rate simultaneously. The steps involved in NSGA-II for minimizing the required objective functions are as follows:

- Initialized random population pop for $F_{pq}$ .
- Evaluated objective functions $O_{i}$ . The first objective function $O_{1}$ considered in this study is the minimum number of features required for the classification of non-ictal and ictal patterns. This is set randomly.
- The second objective function $O_{2}$ considered is the mean error rate Er after 10 iterations of randomized sub-sampling cross-validation. The calculation of Er and the randomized sub-sampling procedure are illustrated as follows:
  - Error rate $\left(Er\right)$ : In this study, the Er corresponds to the classification error for the segregation of non-ictal and ictal patterns. It is given by Fawcett (2006).

(6) Er=100−CA

Here, CA is the mean classification accuracy calculated using equation (7).


(7) $$CA=\left[\left(TN+TP\right)/\left(FP+FN+TN+TP\right)\right]\times 100\%$$

Where TP represents true positive values,

TN represents true negative values,

FP represents false positive values, and

FN represents false negative values.

- Cross-validation by randomized sub-sampling: It is a statistical cross-validation procedure in which the original input data are randomly subdivided into training and testing sets. This process was iterated 10 times, and an equal number of training and testing sets were formed. During each iteration, the CA of the model was evaluated. Here, a generalized regression neural network (GRNN) (illustrated in the next section) was employed for classification. Finally, the mean CA (in %) was subtracted from 100 to evaluate the measure of mean Er (in %), which formed the second objective of this study.

- Applied non-dominated sorting (NDS) to sort the pop . Each chromosome $\left(pop_{1},pop_{2},pop_{3},\ldots .pop_{n}\right)$ in the population was assigned rank along with its crowding distance. The crowding distance is the Euclidean distance between each individual in the front based on objectives.
- Performed selection based on the crowded comparison operator $\left({<}_{n}\right)$ .
- Generated offspring population popc using cross-over and mutation operations (Heris, 2015).
- Evaluated objective functions for popc . During this process, the offspring population popc are combined with the current pop .
- The NDS was again applied and the selection of the individuals for the next iteration was performed based on rank and the crowding distance assigned.
- The next iteration is filled subsequently by each Pareto front. If by adding all the elements from a Pareto front, population size exceeds p (i.e., number of signals), then individuals from that Pareto front are taken based on crowding distance in descending order till population size reaches p .
- Steps iv–ix are repeated until the algorithm converges.
- Once the algorithm converges, the Pareto front is made based on the chromosome’s rank and crowding distance. The solution is achieved based on tournament selection.

**Figure 5:**
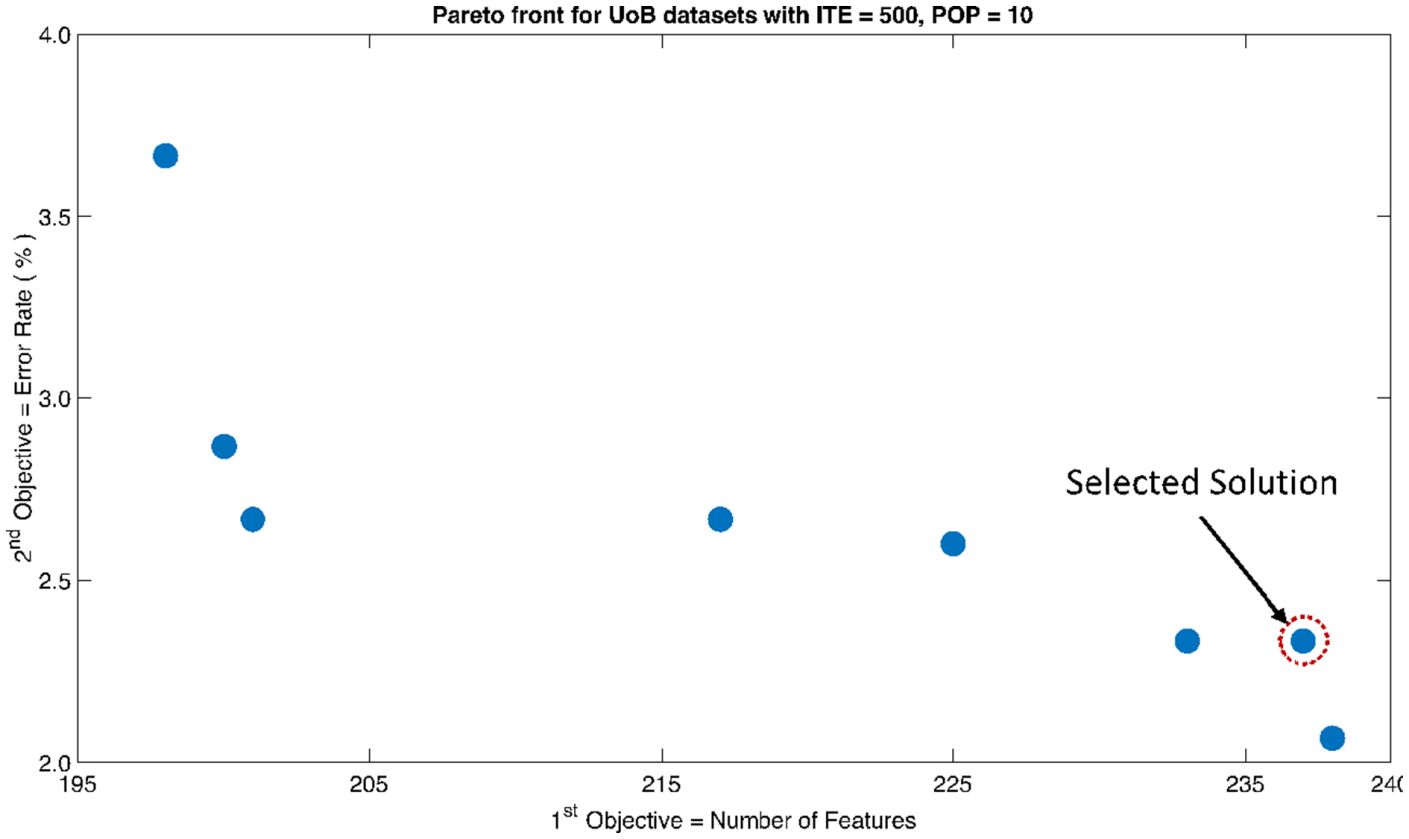
Non-dominant Solutions for University of Bonn (UoB) datasets when subjected to 500 iterations (ITE) and 10 population size (POP).

**Figure 6:**
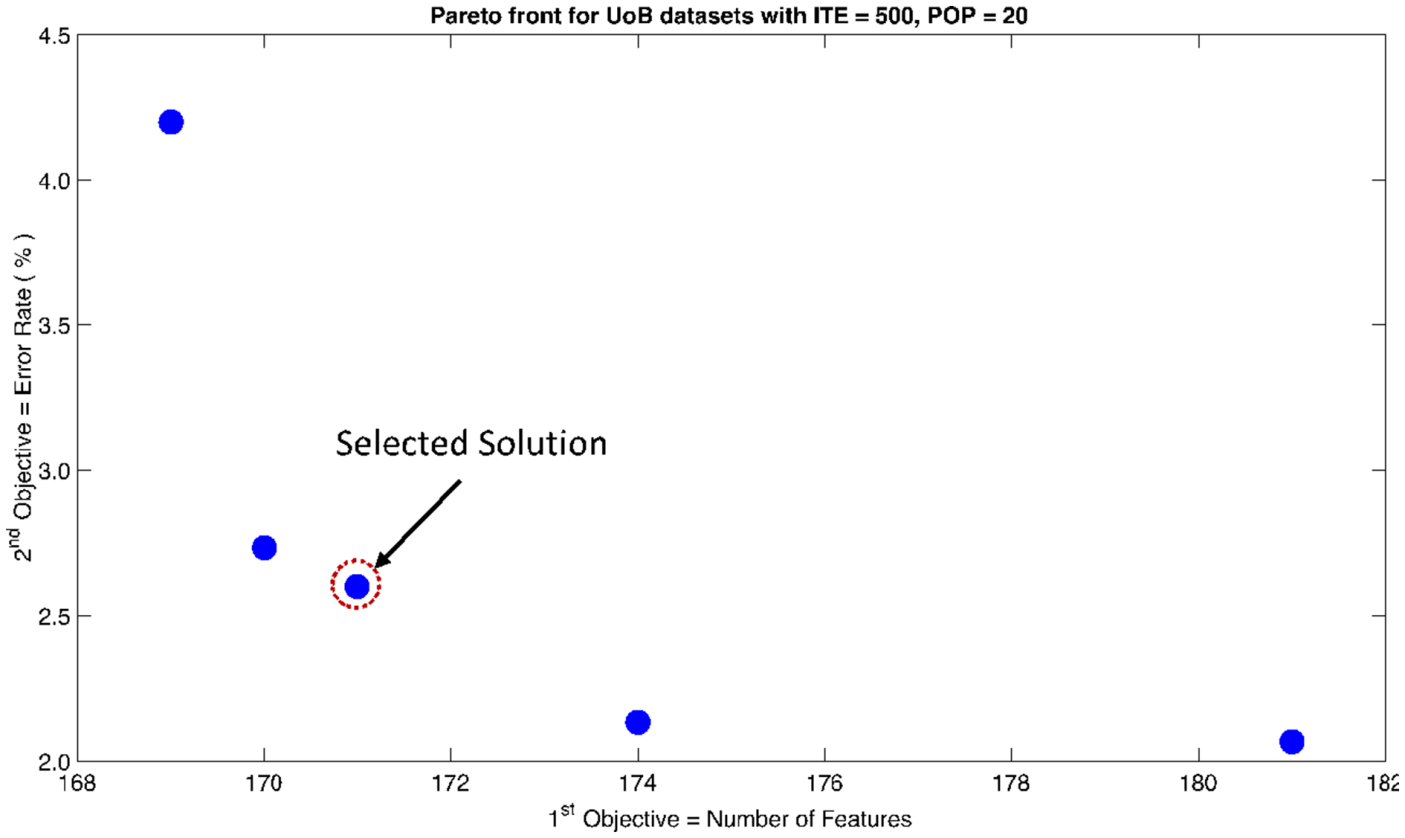
Non-dominant Solutions for University of Bonn (UoB) datasets when subjected to 500 iterations (ITE) and 20 population size (POP).

**Figure 7:**
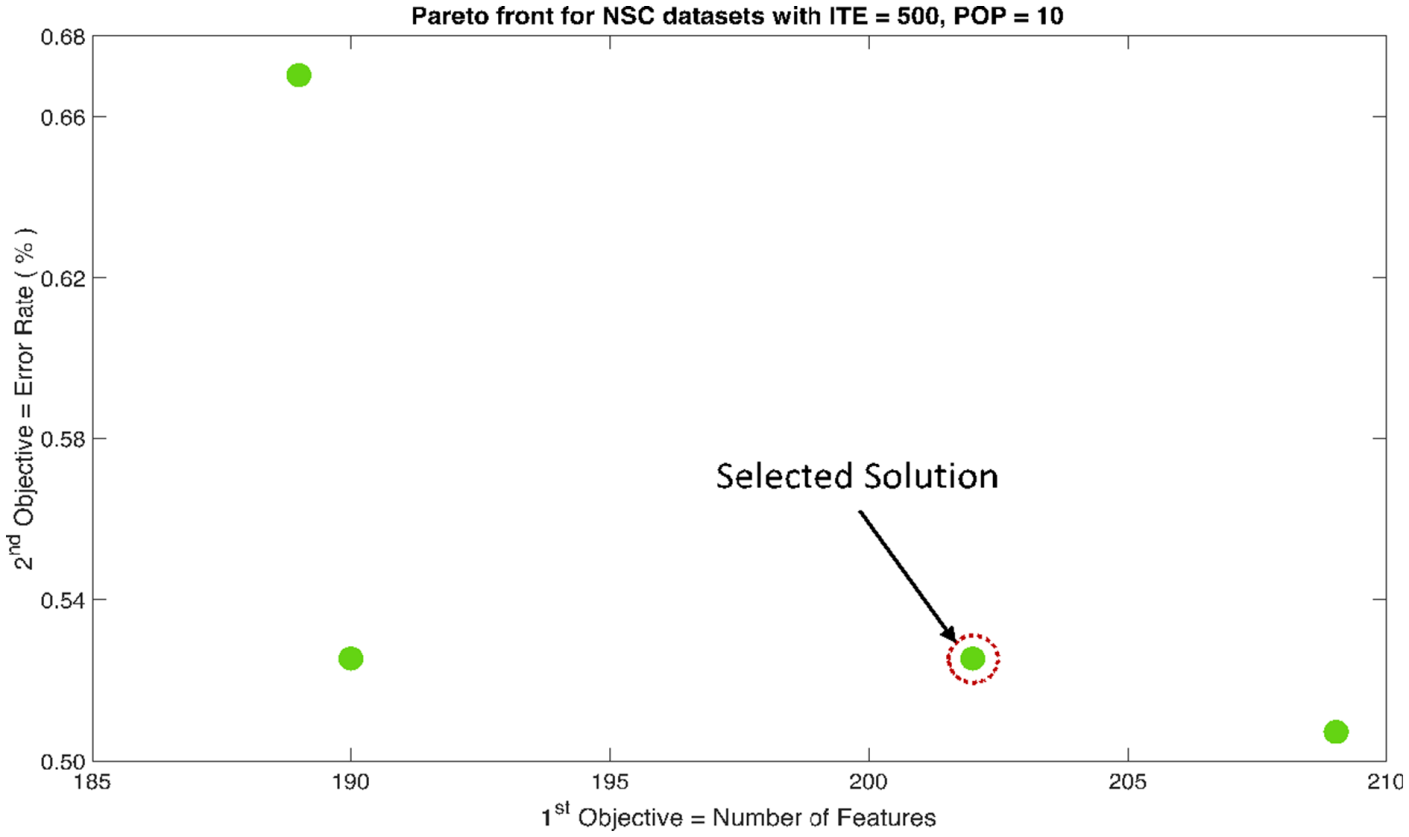
Non-dominant Solutions for Neurology & Sleep Centre (NSC) datasets when subjected to 500 iterations (ITE) and 10 population size (POP).

**Figure 8:**
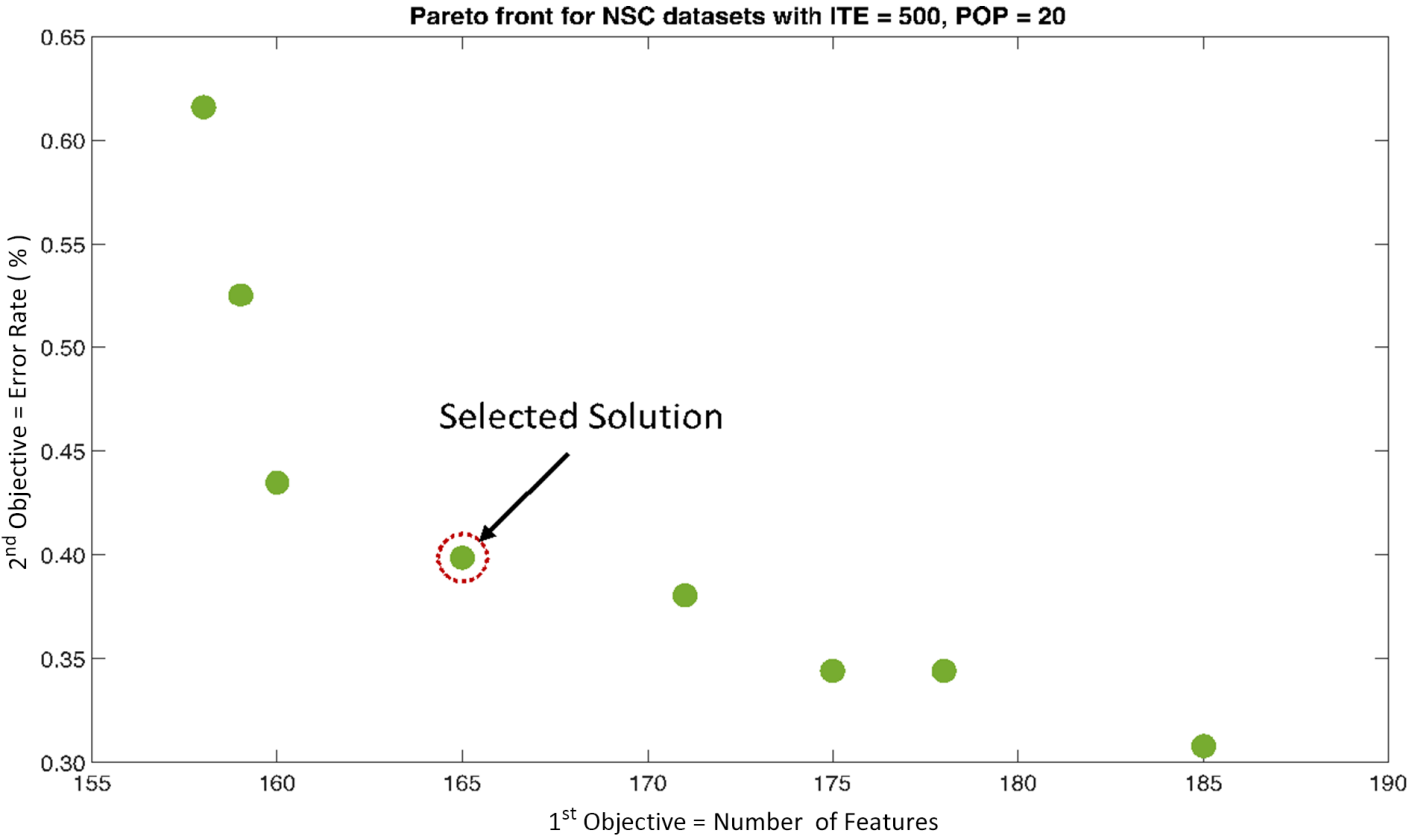
Non-dominant Solutions for Neurology & Sleep Centre (NSC) datasets when subjected to 500 iterations (ITE) and 20 population size (POP).

**Figure 9:**
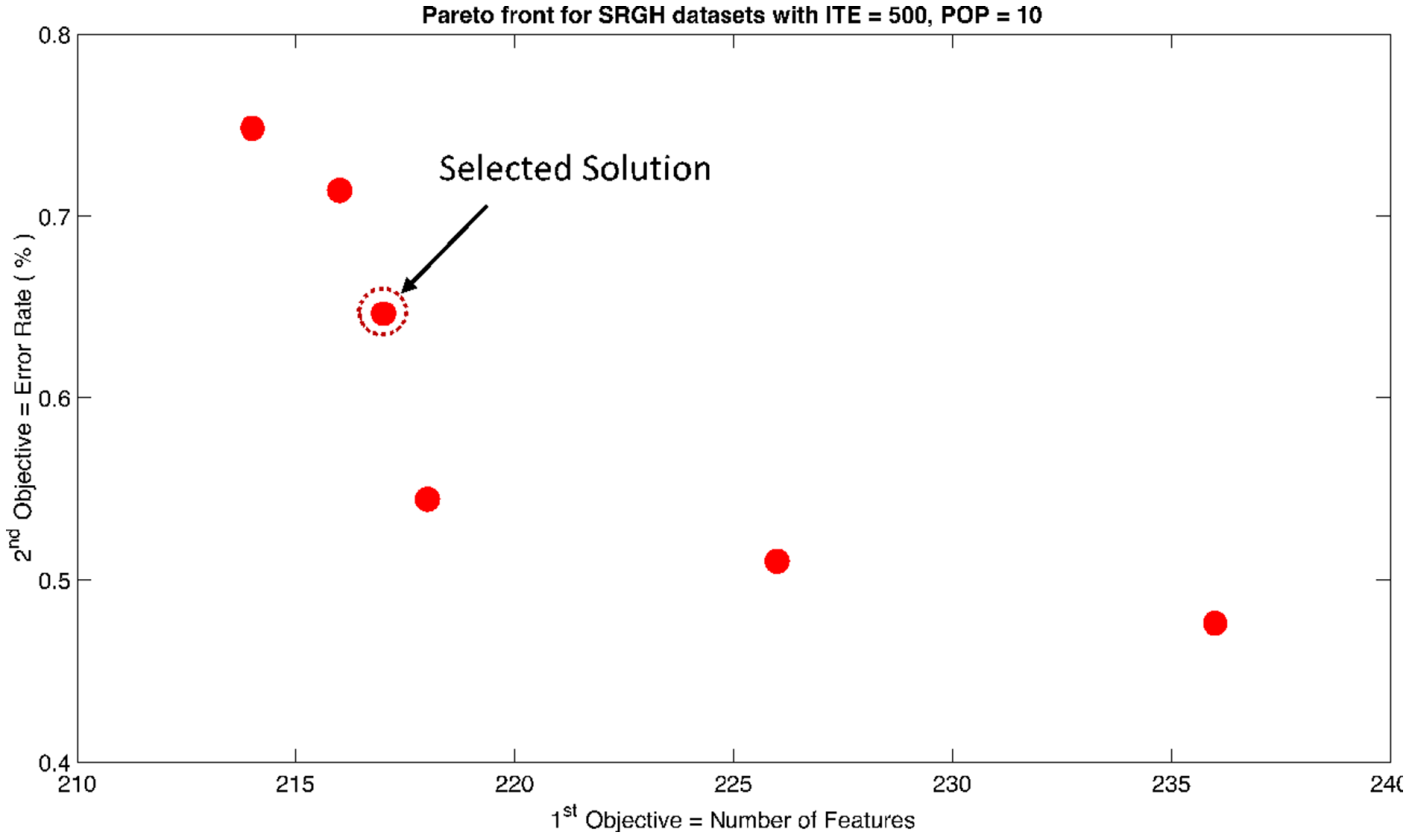
Non-dominant Solutions for Sri Ganga Ram Hospital (SRGH) datasets when subjected to 500 iterations (ITE) and 10 population size (POP).

**Figure 10:**
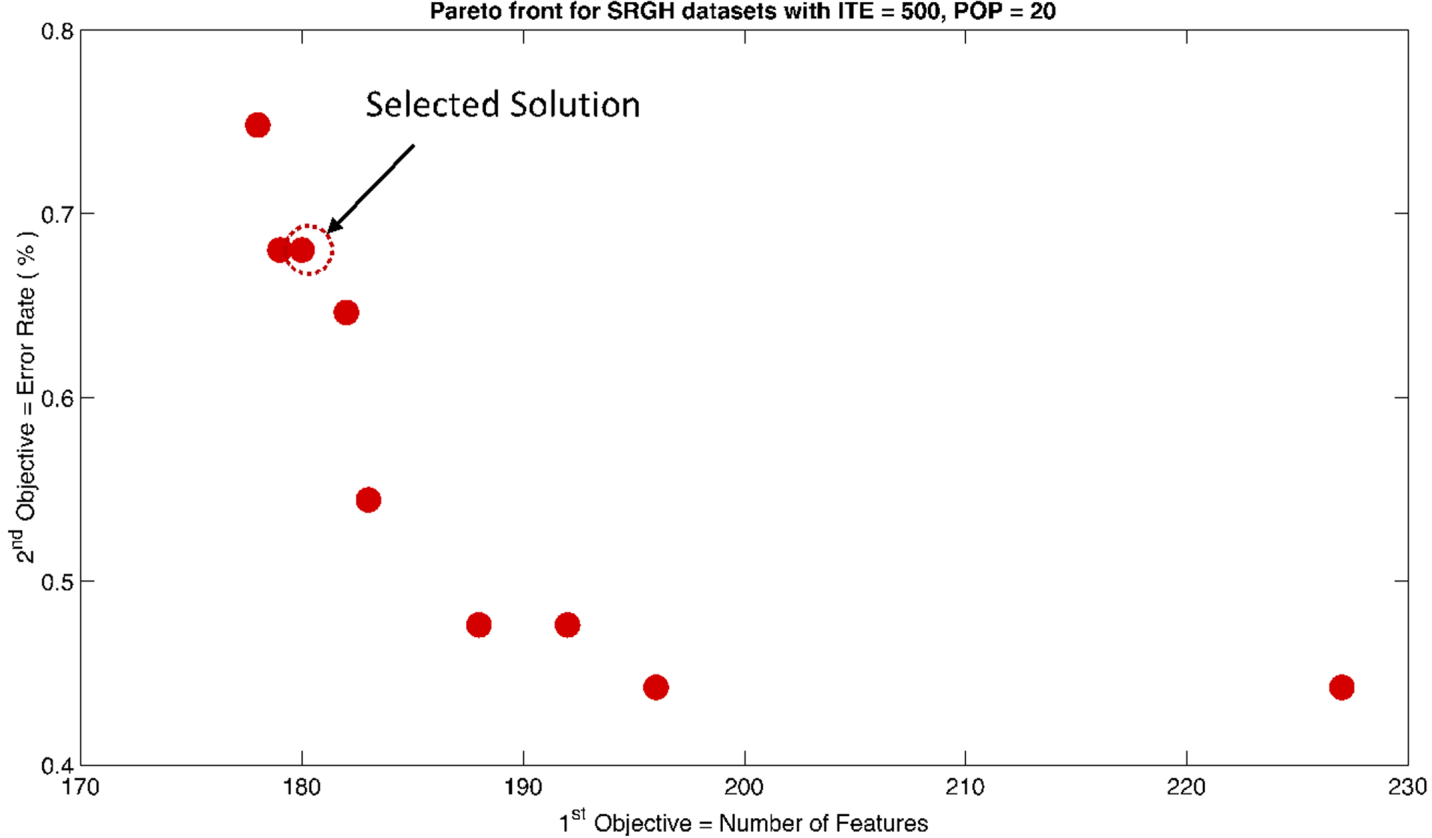
Non-dominant Solutions for Sri Ganga Ram Hospital (SRGH) datasets when subjected to 500 iterations (ITE) and 20 population size (POP).

The rank 1 Pareto front of the University of Bonn (UoB) datasets is depicted in Figures 5, 6. Figure 5 resulted from subjecting the NSGA-II method to 500 iterations with a population size of 10, while Figure 6 was generated using the same method but with a population size of 20. The selected solution after step xi is highlighted in both Figures 5, 6. Similarly, the results of the rank 1 Pareto front for the Neurology & Sleep Centre (NSC) datasets are presented in Figures 7, 8. Additionally, the results for the Sri Ganga Ram Hospital (SGRH) datasets are shown in Figures 9, 10. Figures 7, 9 were obtained when the NSGA-II method underwent 500 iterations with a population size of 10, whereas Figures 8, 10 were generated with a population size of 20 under the same method.

### 2.4 Generalized regression neural network (GRNN) based classification

In artificial intelligence (AI), the classifier usually maps the function of input feature space to output class space. This could be mathematically expressed as $f:R^{a}\to R^{b}$ , where f represents the function, a is the dimension of input feature space, and b is the dimension of output feature. In neural networks, this mapping is achieved by the simulation of artificial neural clusters, such as the human brain. The GRNN model predicts the output/target class by predicting the probability density functions of the input data. GRNN has a memory-based architecture, and the solution is converged to the regression surface by following an asymptotic curve (Specht, 1991; Tomand and Schober, 2001). The parallel and one-pass learning architecture of the GRNN model is a lot faster than the recurrent neural networks.

**Figure 11:**
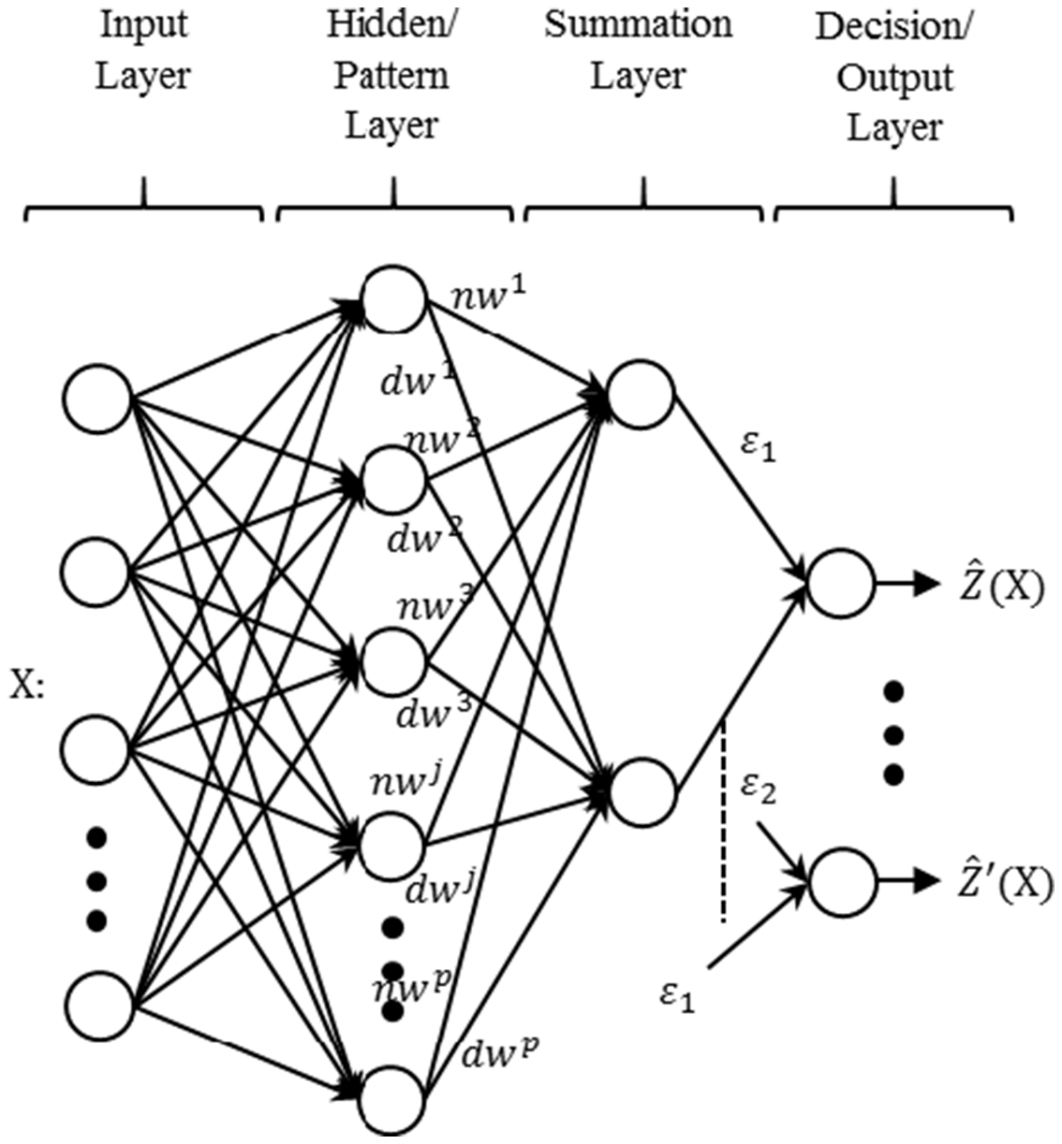
Typical architecture of generalized regression neural network (GRNN).

The typical architecture of GRNN could be divided into four layers (shown in Figure 11). The input layer is the first layer of the GRNN model, which distributes the input data X among all the neurons after scaling. The second layer is the pattern or hidden layer. It applies the radial basis function to the probability density estimates. The spread of the radial basis function follows a Gaussian-shaped curve and is directly dependent on the value of the smoothing parameter σ . The measured values are passed into the next layer’s neurons, which consists of the summation units (one in the denominator and the other in the numerator). The denominator unit sums input weights $dw_{j}$ for all the samples from the pattern layer’s neurons. Similarly, the numerator unit sums the weights $nw_{j}$ for all the samples with actual targets of the pattern layer’s neurons. The final values of the numerator and the denominator have been indicated by $\varepsilon_{1}$ and $\varepsilon_{2}$ , respectively. The final layer of the GRNN model acts like an accumulator, which divides the $\varepsilon_{1}$ and $\varepsilon_{2}$ inputs to predict the output value $\hat{Z}\left(X\right)$ . This value could also be expressed by assuming function $f\left(X,Y\right)$ as the probability density of random variables X and Y . The density estimation is $\hat{f}\left(X,Y\right)$ for samples ${Χ}^{i}$ and $Y^{i}\text{,}$ where i is the indices of the samples. The final predicted targets $\hat{Y}\left(Χ\right)$ for p number of signals is given by equation (8).


(8) $$\hat{Y}\left(X\right)=\overset{p}{\underset{i=1}{\sum }}Y^{i}e^{\frac{-\delta_{i}^{2}}{2\sigma^{2}}}/\overset{p}{\underset{i=1}{\sum }}e^{\frac{-\delta_{i}^{2}}{2\sigma^{2}}}$$

Where scaling function $\delta_{i}^{2}={\left(Χ-{Χ}^{i}\right)}^{T}\left(Χ-{Χ}^{i}\right).$

Based on equation (6), the outputs are similarly updated for ${\hat{Y}}^{\prime }\left(Χ\right)$ values.

Performance parameters: The following parameters were considered for evaluating the performance of the expert system developed:

Classification Accuracy (CA): It is the measure of the expert system to correctly classify the signals in the testing set as given by equation (7).

Sensitivity (SN): It is the statistical measure of the expert system to correctly classify the ictal patterns in EEG. It is evaluated by equation (9).


(9) $$SN=\left[\frac{TP}{FN+TP}\right]\times 100\%$$

Specificity (SP): It is the statistical measure of the expert system to correctly classify the non-ictal patterns in EEG. It is evaluated by equation (10).


(10) $$SP=\left[\frac{TN}{FP+TN}\right]\times 100\%$$

Mathew’s Correlation Coefficient (MCC): It is a balanced statistical measure that considers both the sensitivity and specificity values of the expert system. It is calculated using equation (11) (Jurman et al., 2012). The value varies from -1 to 1. The closer the value of MCC toward 1, the better the prediction (Jurman et al., 2012).


(11) $$MCC=\frac{\left\{\left(TP\times TN\right)-\left(FP\times FN\right)\right\}}{\sqrt{\left(TP+FP\right)\left(TP+FN\right)\left(TN+FP\right)\left(TN+FN\right)}}$$

Computation Time (CT): It is the measure of the total time elapsed for classifying the signals in the testing set. In this study, CT was measured in s.

## 3 Results and discussion

In this study, datasets from three different repositories were evaluated for the classification of interictal (non-ictal) versus ictal patterns. Each dataset was decomposed into WPT coefficients, and various types of features were extracted, resulting in a total of 512 features. These features underwent EMO using the NSGA-II method. The unoptimized and optimized feature sets were then inputted into the GRNN ML classifier. Performance parameters, including CA, SN, SP, MCC, and CT, were extracted. Subsequently, a one-way analysis of variance (ANOVA) was conducted across the results of each dataset.

In each of Tables 1–3, $OF_{1}$ represents the optimum features selected for the specific dataset when NSGA-II was subjected to 500 iterations and a population size of 10. Similarly, $OF_{2}$ denotes the optimum features selected for the specific dataset when NSGA-II was subjected to 500 iterations and a population size of 20.

**Table 1:** Classification results using University of Bonn (UoB) datasets.

Results using University of Bonn (UoB) datasets							
Features	$O_{1}$	$O_{2}$ (%)	CA (%)	SN (%)	SP (%)	MCC	CT (s)
$F_{1}F_{2}F_{3}F_{4}$	512	5.67	94.33 ± 1.26	90.62 ± 5.75	96.91 ± 2.14	0.88 ± 0.03	0.028 ± 0.001
$OF_{1}$	237	2.33	97.67 ± 1.57^***^	96.03 ± 1.64	96.71 ± 2.04	0.92 ± 0.03	0.027 ± 0.001
$OF_{2}$	171	2.60	97.40 ± 0.83	96.64 ± 2.34^***^	97.66 ± 1.64	0.94 ± 0.02^***^	0.028 ± 0.002

***means *p* < 0.001; **means *p* < 0.01; *means *p* < 0.05; ns means *p*-value is not significant.

**Table 2:** Classification results using Neurology & Sleep Centre (NSC) datasets.

Results using Neurology & Sleep Centre (NSC) datasets							
Features	$O_{1}$	$O_{2}$ (%)	CA (%)	SN (%)	SP (%)	MCC	CT (s)
$F_{1}F_{2}F_{3}F_{4}$	512	1.36	98.64 ± 0.56	97.93 ± 0.76	99.40 ± 0.56	0.97 ± 0.01	0.475 ± 0.001
$OF_{1}$	202	0.52	99.48 ± 0.31	99.24 ± 0.39	99.46 ± 0.52	0.99 ± 0.01^**^	0.475 ± 0.001
$OF_{2}$	165	0.40	99.60 ± 0.28^**^	98.99 ± 0.52^***^	99.55 ± 0.38	0.98	0.482 ± 0.006

***means *p* < 0.001; **means *p* < 0.01; *means *p* < 0.05; ns means *p*-value is not significant.

**Table 3:** Classification results using Sri Ganga Ram Hospital (SGRH) datasets.

Results using Sri Ganga Ram Hospital (SGRH) datasets							
Features	$O_{1}$	$O_{2}$ (%)	CA (%)	SN (%)	SP (%)	MCC	CT (s)
$F_{1}F_{2}F_{3}F_{4}$	512	1.56	98.43 ± 0.27	98.22 ± 1.25	98.65 ± 1.26	0.97 ± 0.08	0.072 ± 0.000
$OF_{1}$	216	0.71	99.29 ± 0.02	98.75 ± 1.22	99.47 ± 0.82	0.98 ± 0.02	0.072 ± 0.001
$OF_{2}$	180	0.68	99.32 ± 0.51	98.96 ± 0.99	99.10 ± 1.03	0.98 ± 0.02	0.072 ± 0.000

***means *p* < 0.001; **means *p* < 0.01; *means *p* < 0.05; ns means *p*-value is not significant.

Table 1 presents the classification results of the datasets collected from the UoB database. A highly significant (*p* < 0.001) CA of 97.67 ± 1.57% was achieved using $OF_{1}$ features from the UoB datasets. When the features were reduced to 171, a significantly high (*p* < 0.001) SN of 96.64 ± 2.34% was observed for $OF_{2}$ . Furthermore, the MCC values were also highly significant (*p* < 0.001), measuring 0.94 ± 0.02 with $OF_{2}$ features.

The feature matrix $OF_{2}$ of NSC datasets was significantly reduced to only 165 features, yet the optimally selected feature sets produced significant results across CA and SN (Table 2). However, the MCC measure of $OF_{1}$ proved to be more significant (*p* < 0.01) with a value of 0.99 ± 0.01.

In contrast, the results in Table 3 did not yield any significant outcomes. Nevertheless, the optimally selected features were reduced to 216 with an error rate of 0.71% for $OF_{1}$ and 180 features with an error rate of 0.68% for $OF_{1}$ .

Overall, the results indicate that the optimized features demonstrated significant or comparable performance to the complete feature sets. It was observed that $OF_{2}$ features across all combinations were maximally reduced, suggesting that an increase in population size with the same iterations further reduces the feature sets.

## 4 Conclusion and future scope

This study successfully demonstrated the classification of interictal versus ictal patterns across three different datasets, achieving the objectives proposed in the introduction section. The computation time during all tests was less than 0.5 s, showcasing the applicability of the proposed expert system for real-time clinical settings. To ensure transparency of the expert system, the proven biological relevance for choosing each of the features extracted in this study was discussed along with the main mathematical formula. This also aimed to develop clinicians’ trust and adaptation to AI tools for future assistance.

A significant novelty of this study is the successful and methodical demonstration of ETO (Tan et al., 2021) for epilepsy diagnosis. While most existing literature achieves similar accuracy using only publicly available datasets, this study incorporates results from both public and private repositories, ensuring the generalization of the expert system. The MAMF concept assures the scalability of the expert system.

In the future, increasing the number of multi-objective functions to “many” could enhance performance and, importantly, the generalizability of expert systems. For example, this could be achieved by using NSGA-III. Additional objectives to consider may include improving statistical performance (Mormann et al., 2007; Tiwari et al., 2016; Swami et al., 2019; Anuragi et al., 2022) while further reducing the number of channels required for diagnosis. This could be extended with deep learning (DL) methods (Tang et al., 2024) and/or localizing the foci of epileptic seizures, thus addressing long-standing inverse problems (Swami et al., 2016c,d; Gandhi et al., 2024). This study was conducted using three datasets (as described in section 3.1); however, the total number of participants across all three datasets was 35 and the brain signals as annotated by clinicians and thereby classified using the proposed method were inter-ictal vs. ictal pattern recognition. Our group is also working on in-house annotation of signals collected from more participants and their real-time classification of pre-ictal patterns. This would be another class for the upgraded expert system. Furthermore, to increase the generalization and effectiveness of the model to detect different types of seizure patterns and non-epileptic clinical conditions manifesting seizure-like patterns, the future scope also includes annotation of such types of patterns and testing on continuous long-term brain signal recordings.

## Data availability statement

The datasets presented in this study can be found in online repositories. The names of the repository/repositories and accession number(s) can be found below: https://www.researchgate.net/publication/308719109_EEG_Epilepsy_Datasets.

## Author contributions

PS: Conceptualization, Data curation, Methodology, Writing – original draft. JM: Conceptualization, Data curation, Writing – original draft. MK: Writing – review & editing. MB: Data curation, Validation, Writing – review & editing.

## References

[ref1] Acharya U. R. Vinitha Sree S. Swapna G. Martis R. J. Suri J. S. (2013). Automated EEG analysis of epilepsy: a review. Knowl. Based Syst. 45, 147–165. doi: 10.1016/j.knosys.2013.02.014

[ref2] Andrzejak R. G. Lehnertz K. Mormann F. Rieke C. David P. Elger C. E. (2001). Indications of nonlinear deterministic and finite-dimensional structures in time series of brain electrical activity: dependence on recording region and brain state. Phys. Rev. E Stat. Nonlinear Soft Matter Phys. 64:061907. doi: 10.1103/PhysRevE.64.061907, PMID: 11736210

[ref3] Anuragi A. Sisodia D. S. Pachori R. B. (2022). Epileptic-seizure classification using phase-space representation of FBSE-EWT based EEG sub-band signals and ensemble learners. Biomed Signal Process. Control 71:103138. doi: 10.1016/j.bspc.2021.103138

[ref4] Banerjee P. N. Filippi D. Hauser W. A. (2009). The descriptive epidemiology of epilepsy-a review. Epilepsy Res. 85, 31–45. doi: 10.1016/j.eplepsyres.2009.03.003, PMID: 19369037 PMC2696575

[ref5] Behara D. S.T., Kumar A. Swami P., Panigrahi B. K., & Gandhi T. K. (2016). Detection of epileptic seizure patterns in EEG through fragmented feature extraction. New Delhi, India: 3rd IEEE International Conference on Computing for Sustainable Global Development (INDIACom) (pp. 2539–2542).

[ref6] Dash D. P. Kolekar M. Chakraborty C. Khosravi M. R. (2024). Review of machine and deep learning techniques in epileptic seizure detection using physiological signals and sentiment analysis. ACM Trans. Asian Low Resour. Lang. Inform. Process. 23, 1–29. doi: 10.1145/3552512

[ref7] Deb K. (2001). Multi-objective optimization using evolutionary algorithms, vol. 16: John Wiley & Sons.

[ref8] Dhiman R. Saini J. S. (2014). Genetic algorithms tuned expert model for detection of epileptic seizures from EEG signatures. Appl. Soft Comput. 19, 8–17. doi: 10.1016/j.asoc.2014.01.029

[ref9] Duque-muñoz L. Espinosa-oviedo J. J. Castellanos-dominguez C. G. (2014). Identification and monitoring of brain activity based on stochastic relevance analysis of short – time EEG rhythms. Biomed. Eng. Online 13, 123–120. doi: 10.1186/1475-925X-13-123 25168571 PMC4459461

[ref10] Edakawa K. Yanagisawa T. Kishima H. Fukuma R. Oshino S. Khoo H. M.. (2016). Detection of epileptic seizures using phase–amplitude coupling in intracranial electroencephalography. Sci. Rep. 6:25422. doi: 10.1038/srep25422, PMID: 27147119 PMC4857088

[ref11] Faust O. Acharya U. R. Adeli H. Adeli A. (2015). Wavelet-based EEG processing for computer-aided seizure detection and epilepsy diagnosis. Seizure 26, 56–64. doi: 10.1016/j.seizure.2015.01.012, PMID: 25799903

[ref12] Fawcett T. (2006). An introduction to ROC analysis. Pattern Recogn. Lett. 27, 861–874. doi: 10.1016/j.patrec.2005.10.010

[ref13] Frølich L. Andersen T. S. Mørup M. (2015). Classification of independent components of EEG into multiple artifact classes. Psychophysiology 52, 32–45. doi: 10.1111/psyp.12290, PMID: 25048104

[ref14] Gajic D. Djurovic Z. Gligorijevic J. Di Gennaro S. Savic-Gajic I. (2015). Detection of epileptiform activity in EEG signals based on time-frequency and non-linear analysis. Front. Comput. Neurosci. 9:38. doi: 10.3389/fncom.2015.00038 25852534 PMC4371704

[ref15] Gandhi T. K. Chakraborty P. Roy G. G. Panigrahi B. K. (2012). Discrete harmony search based expert model for epileptic seizure detection in electroencephalography. Expert Syst. Appl. 39, 4055–4062. doi: 10.1016/j.eswa.2011.09.093

[ref16] Gandhi T. Panigrahi B. K. Anand S. (2011). A comparative study of wavelet families for EEG signal classification. Neurocomputing 74, 3051–3057. doi: 10.1016/j.neucom.2011.04.029

[ref17] Gandhi T. Panigrahi B. K. Bhatia M. Anand S. (2010). Expert model for detection of epileptic activity in EEG signature. Expert Syst. Appl. 37, 3513–3520. doi: 10.1016/j.eswa.2009.10.036

[ref18] Gandhi T. K. Swami P. Panigrahi B. K. (2024). A device for automated diagnosis of epilepsy. Indian patent number 500189 with application number 201611043166 granted on Jan. Indian Patent.

[ref19] Gotman J. (1999). Automatic detection of seizures and spikes. Clin. Neurophysiol. 16, 130–140. doi: 10.1097/00004691-199903000-00005 10359498

[ref20] Heris M. K. (2015). NSGA-II in MATLAB, Yarpiz. Available at: https://yarpiz.com/56/ypea120-nsga2.

[ref21] Jurman G. Riccadonna S. Furlanello C. (2012). A comparison of MCC and CEN error measures in multi-class prediction. PLoS One 7:e41882. doi: 10.1371/journal.pone.0041882 22905111 PMC3414515

[ref22] Krishnan P. T. Erramchetty S. K. Balusa B. C. (2024). Advanced framework for epilepsy detection through image-based EEG signal analysis. Front. Hum. Neurosci. 18:1336157. doi: 10.3389/fnhum.2024.1336157, PMID: 38317649 PMC10839025

[ref23] Li D. Xie Q. Jin Q. Hirasawa K. (2016). A sequential method using multiplicative extreme learning machine for epileptic seizure detection. Neurocomputing 214, 692–707. doi: 10.1016/j.neucom.2016.06.056

[ref24] Mormann F. Andrzejak R. G. Elger C. E. Lehnertz K. (2007). Seizure prediction: the long and winding road. Brain 130, 314–333. doi: 10.1093/brain/awl241 17008335

[ref25] Moshé S. L. Perucca E. Ryvlin P. Tomson T. (2015). Epilepsy: new advances. Lancet 385, 884–898. doi: 10.1016/S0140-6736(14)60456-6 25260236

[ref26] Nara S. Swami P. Bhatia M. Panigrahi B. K. Gandhi T. (2016). Efficient recognition of ictal activities in EEG through correlation based dimensionality reduction. New Delhi, India: 3rd IEEE international conference on computing for sustainable global development (INDIACom) (pp. 2547–2550).

[ref27] Pascual D. Amirshahi A. Aminifar A. Atienza D. Ryvlin P. Wattenhofer R. (2020). EpilepsyGAN: synthetic epileptic brain activities with privacy preservation. IEEE Trans. Biomed. Eng. 68, 2435–2446. doi: 10.1109/TBME.2020.3042574 33275573

[ref28] Polat K. Güneş S. (2007). Classification of epileptiform EEG using a hybrid system based on decision tree classifier and fast Fourier transform. Appl. Math. Comput. 187, 1017–1026. doi: 10.1016/j.amc.2006.09.022

[ref29] Schuyler R. White A. Staley K. Cios K. J. (2007). Epileptic seizure detection. IEEE Eng. Med. Biol. Mag. 26, 74–81. doi: 10.1109/MEMB.2007.335592 17441611

[ref30] Sharma R. Pachori R. B. (2015). Classification of epileptic seizures in EEG signals based on phase space representation of intrinsic mode functions. Expert Syst. Appl. 42, 1106–1117. doi: 10.1016/j.eswa.2014.08.030

[ref31] Smith A. E. (2002). Multi-objective optimization using evolutionary algorithms [book review]. IEEE Trans. Evol. Comput. 6, 526–520. doi: 10.1109/TEVC.2002.804322

[ref32] Specht D. F. (1991). A general regression neural network. IEEE Trans. Neural Netw. 2, 568–576. doi: 10.1109/72.97934 18282872

[ref33] Srinivasan S. Dayalane S. Mathivanan S. K. Rajadurai H. Jayagopal P. Dalu G. T. (2023). Detection and classification of adult epilepsy using hybrid deep learning approach. Sci. Rep. 13:17574. doi: 10.1038/s41598-023-44763-7, PMID: 37845403 PMC10579259

[ref34] Swami P. Anand S. Panigrahi B. K. (2015c). Automatic detection of epileptiform signatures in electroencephalography. Melbourne, Australia: Proc. 7th Int. workshop on seizure prediction.

[ref35] Swami P. Bhatia M. Anand S. Panigrahi B. K. Santhosh J. (2015a). SVM based automated EEG seizure detection using ‘Coiflets’ wavelet packets. Noida, India: IEEE 2015 International Conference on Recent Developments in Control, Automation and Power Engineering (RDCAPE) (pp. 238–242).

[ref36] Swami P. Bhatia M. Tripathi M. Chandra P. S. Panigrahi B. K. Gandhi T. K. (2019). Selection of optimum frequency bands for detection of epileptiform patterns. IEEE IET Healthcare Technol. Lett. 6, 126–131. doi: 10.1049/htl.2018.5051, PMID: 31839968 PMC6849498

[ref37] Swami P. Gandhi T. Panigrahi B. K. Bhatia M. (2016b). ‘Epilepsy Datasets’: Research Gate. Available at https://www.researchgate.net/publication/308719109_EEG_Epilepsy_Datasets.

[ref38] Swami P. Gandhi T. Panigrahi B. K. Bhatia M. Anand S. (2015b). Detection of ictal patterns in electroencephalogram signals using 3D phase trajectories. IEEE proceedings of annual India international conference, New Delhi, India. (pp. 1–6).

[ref39] Swami P. Gandhi T. Panigrahi B. K. Bhatia M. Anand S. (2016c). Locating ictal activities over human scalp with automated detection using EEG signals. IEEE proceedings of international conference on signal processing and integrated networks, Noida, India. (pp. 1–6).

[ref40] Swami P. Gandhi T. K. Panigrahi B. K. Bhatia M. Santhosh J. Anand S. (2017). A comparative account of modelling seizure detection system using wavelet techniques. Int. J. Syst. Sci.: Oper. Logist. 4, 41–52. doi: 10.1080/23302674.2015.1116637

[ref41] Swami P. Gandhi T. Panigrahi B. K. Tripathi M. Anand S. (2016a). A novel robust diagnostic model to detect seizures in electroencephalography. Expert Syst. Appl. 56, 116–130. doi: 10.1016/j.eswa.2016.02.040

[ref42] Swami P. Godiyal A. K. Santhosh J. Panigrahi B. K. Bhatia M. Anand S. (2014). Robust expert system design for automated detection of epileptic seizures using SVM classifier. Solan, India: IEEE international conference on parallel, distributed and grid computing (pp. 219–222).

[ref43] Swami P. Panigrahi B. K. Bhatia M. Anand S. Tripathi M. Gandhi T. K. (2016d). Detection and cortical localization of ictal signatures using electroencephalogram signals. Reading, United Kingdom: Proc. INCF World Congr. Neuroinf, 1–2.

[ref44] Swami P. Panigrahi B. K. Bhatia M. Anand S. Tripathi M. Gandhi T. K. (2018). Application of machine learning in seizure detection. Montréal, Canada: Proc. INCF World Congr. Neuroinf.

[ref45] Tan K. C. Feng L. Jiang M. (2021). Evolutionary transfer optimization-a new frontier in evolutionary computation research. IEEE Comput. Intell. Mag. 16, 22–33. doi: 10.1109/MCI.2020.3039066

[ref46] Tang Y. Wu Q. Mao H. Guo L. (2024). Epileptic seizure detection based on path signature and bi-LSTM network with attention mechanism. IEEE Trans. Neural Syst. Rehabil. Eng. 32, 304–313. doi: 10.1109/TNSRE.2024.3350074, PMID: 38224524

[ref47] Tirumani P. V. Das S. Swami P. Gandhi T. (2018). A low-noise low-cost EEG amplifier for neural recording applications. Advanced computational and communication paradigms: Proceedings of international conference on ICACCP 2017. 1 (pp. 581–589). Springer: Singapore.

[ref48] Tiwari A. K. Pachori R. B. Kanhangad V. Panigrahi B. K. (2016). Automated diagnosis of epilepsy using key-point-based local binary pattern of EEG signals. IEEE J. Biomed. Health Inform. 21, 888–896. doi: 10.1109/JBHI.2016.2589971, PMID: 27416609

[ref49] Tomand D. Schober A. (2001). A modified general regression neural network (MGRNN) with new, efficient training algorithms as a robust “black box”-tool for data analysis. Neural Netw. 14, 1023–1034. doi: 10.1016/S0893-6080(01)00051-X, PMID: 11681748

[ref50] Tzallas A. T. Tsipouras M. G. Fotiadi D. I. (2009). Epileptic seizure detection in EEGs using time-frequency analysis. IEEE Trans. Inf. Technol. Biomed. 13, 703–710. doi: 10.1109/TITB.2009.2017939 19304486

[ref51] World Health Organization (2024). Epilepsy. Available at: https://www.who.int/news-room/fact-sheets/detail/epilepsy.

